# Sensor-Based Fall Risk Assessment: A Survey

**DOI:** 10.3390/healthcare9111448

**Published:** 2021-10-27

**Authors:** Guangyang Zhao, Liming Chen, Huansheng Ning

**Affiliations:** 1 School of Computer and Communication Engineering, University of Science and Technology Beijing, Beijing 100089, China; g20198892@xs.ustb.edu.cn; 2 School of Computing, University of Ulster, Newtownabbey BT37 0QB, UK; l.chen@ulster.ac.uk

**Keywords:** fall risk assessment, fall prediction, gait monitoring, sensor

## Abstract

Fall is a major problem leading to serious injuries in geriatric populations. Sensor-based fall risk assessment is one of the emerging technologies to identify people with high fall risk by sensors, so as to implement fall prevention measures. Research on this domain has recently made great progress, attracting the growing attention of researchers from medicine and engineering. However, there is a lack of studies on this topic which elaborate the state of the art. This paper presents a comprehensive survey to discuss the development and current status of various aspects of sensor-based fall risk assessment. Firstly, we present the principles of fall risk assessment. Secondly, we show knowledge of fall risk monitoring techniques, including wearable sensor based and non-wearable sensor based. After that we discuss features which are extracted from sensors in fall risk assessment. Then we review the major methods of fall risk modeling and assessment. We also discuss some challenges and promising directions in this field at last.

## 1. Introduction

Aging population has become a common problem for major countries in the world. Compared with young people, falls are more likely to occur in the elderly. In some countries, such as the United States, falls have become a leading cause of death due to injuries in people over 65 [1]. In addition to physical damage, the elderly people with a history of falls may have greater psychological stress and a narrow scope of daily living activities, resulting in worse quality of life. Furthermore, the resulting personal and social expenditure is a large amount. Therefore, reducing the incidence of falls in the elderly is a matter of great significance. Fall risk assessment is one of the emerging promising technologies for the above goal.

In our opinion, “fall risk” refers to whether a person is prone to falling. This is how most work defines “fall risk” in our reviewed articles. They analyzed the relationship between gait characteristics and fall risk in combination with fall history in the past or fall status in the future. The items “fall detection” and “fall prediction” (fall risk assessment) are often confused. They are protective measures from different perspectives. Fall detection aims at detecting the occurrence of fall event in time so that treatment or protection during fall (e.g., air cells at waist) can be carried out right away. It is an “afterwards approach”. Unlike fall detection, fall risk assessment is a “beforehand approach”. It tries to identify elderly people at high fall risk. Then targeted preventive measures can be taken before the “real fall” happens. This kind of technology has a remarkable social and economic worth. It has received growing attention due to the great progress of sensing, communication and data processing technologies in recent years.

Fall risk assessment is a complicated process based on detection and analysis of factors leading to falls. There are many factors leading to falls, and they have been divided into two categories: external and internal [2]. External factors refer to environmental factors such as room layout, road conditions, to name but a few. Existing studies focus more on internal factors. Internal factors refer to self status, including physical, cognitive and psychological. Older age has been shown to be related to falls, because aging can lead to instability in walking posture [3]. Sarcopenia is a syndrome highly relevant to falls [4]. It is a condition characterized by decreased muscle mass, muscle strength and physical performance. Sensory disturbance is another important factor related to falls [5], such as visual impairment and hearing impairment. In addition, medication, stroke, depression and postural hypotension are also internal factors. According to reviewed papers, there are scale-based and sensor-based approaches to detect and analyze internal factors.

The fall risk scale is an important tool in fall risk assessment. Scale-based assessment is suitable for most internal factors. Researchers fill out the scale through inquiry, observation and measurement. There are many scales for fall risk assessment. The most commonly used are the Berg Balance Scale [6], Tinetti Balance Scale [7], STRATIFY [8], and so on. Different scales are suitable for different situations. For example, STRATIFY was used only for elderly hospitalized patients [9]. Several reviews about scale-based fall risk assessment have been published over the years. A systematic review in 2018 by Park [9] paid attention to the quantitative analysis of the predictive validity of scales. The author pointed out that combining two assessment tools was more effective than using a single tool due to more factors were contained. Another review in 2018 by Ruggieri and colleagues [10] focused entirely on the setting, language, pathology and psychometric properties of scales. Together these papers have provided a comprehensive overview on the scales used in fall risk assessment. Given the existing works, we do not review research on the scale-based approach. It is necessary to point out that, while the scale-based approach is comprehensible and low-cost, it suffers from being subjective.

In recent years, technologies like sensing, efficient wireless communication and data processing have made significant progress. The advances and maturity of above mentioned technologies make a lot of researchers try to realize fall risk assessment by sensors. Sensor-based approaches focus on characteristics of kinematics and kinetics of a person. It tries to assess one’s fall risk through motion state. Compared to the scale-based approach, the sensor-based approach is more objective, and at the same time, easy to implement. Sensor-based fall risk assessment is a complicated process that can be roughly characterized by four steps. These steps include (1) to select and fix wearable sensors to the subjects or deploy non-wearable sensors to environments to monitor the motion of the subjects, (2) to make the participants walk by rules and collect and transmit data that is obtained by the sensors, (3) to preprocess the data collected and choose or develop algorithms to establish models, (4) to use the model in last step to assess the relationship between gait status and fall risk using sensor data as input.

Compared to the number of surveys in scale-based fall risk assessment, there is a lack of comprehensive overviews on the latest development of sensor-based fall risk assessment. Considering this, a systematic survey will be of high value. It can inform the researchers of the current status and future promising directions. This survey aims at presenting a comprehensive overview on the state of the art of sensor-based fall risk assessment. It will cover the life cycle of the approach and provide descriptions and comparisons of various methods to highlight their advantages and disadvantages. In this survey, we review related works based on the order from monitoring to features to modeling and assessment. After the introduction, the organization of this article is as follows. In Section 2, we investigate the monitoring techniques used in sensor-based fall risk assessment. We then discuss about features in sensor-based fall risk assessment in Section 3. In Section 4, We review major modeling techniques and present comparison of these techniques. In Section 5, we provide insights into existing challenges of fall risk assessment. Potential future research directions of this field are listed in Section 6. The survey is concluded in Section 7.

## 2. Fall Risk Monitoring

A wide range of sensors, including inertial sensors like accelerometers and gyroscopes, pressure sensors, and infrared sensors, to name but a few, are used in fall risk assessment. These various sensors have different types, functions, output signals, and technical principles. They can be classified as wearable sensors and non-wearable sensors according to the way they are deployed. Wearable sensors are the most commonly used. In the following we present the common practice in sensor-based fall risk assessment.

### 2.1. Wearable Sensors

Wearable sensors are sensors which are directly or indirectly fixed to human body, and they generate signals when the user moves or performs other activities. Wearable sensors can be embedded into daily objects like belts and shoes or directly fixed to the body. They can monitor movement status or physiological information when properly worn by users.

**Figure 1:**
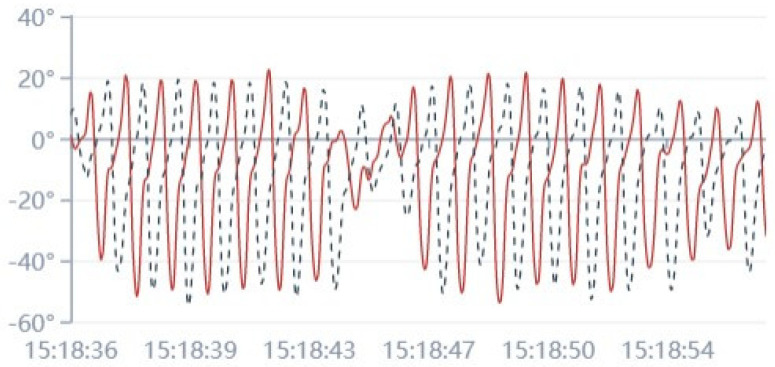
The angle curve of leg flexion and extension in the vertical direction during walking. The red line represents the left leg, and the black dotted line represents the right leg.

Inertial sensors and pressure sensors are the most frequently used wearable sensors in fall risk assessment. Inertial sensors mainly include accelerometers and gyroscopes. They are suitable for monitoring body motions. In Figure 1, we show a graphic example of leg flexion and extension angle during walking, which was obtained in our real application. Generally, inertial sensors are fixed to different body parts to obtain different motion features and pressure sensors are embedded into insoles. Howcroft et al. [11] placed tri-axial accelerometers on head, lower back and left and right shanks of older individuals under single-task and dual-task conditions to identify the optimal sensor combination, placement and modeling approaches for fall risk assessment. In addition, participants were required to wear pressure-sensing insoles. Accelerometer-based features used in this study were maximum, mean, and standard deviation of acceleration for different axes, cadence, stride time, fast Fourier transform (FFT) Quartile, ratio of even to odd harmonics (REOH), and maximum Lyapunov exponent (MLE). For pressure-sensing insoles, they derived features like center of pressure (CoP) path, temporal features such as stride time symmetry index between left and right limbs and impulse from the total force-time curve. The results indicated that multi-layer perceptron had a better performance than naïve Bayesian and support vector machine. In single-task fall risk classification, head sensor-based models had the best performance. Accelerometers were placed on lower limb (ankle, shank, etc.) to obtain spatiotemporal gait features like gait speed [11,12]. Doheny et al. [13] used tri-axial accelerometers on the thigh to record the process from sitting to standing during the five-times-sit-to-stand test. Weiss et al. [14] asked 107 Parkinson’s patients to fix a small three-axis accelerometer to the lower back for three days respectively. Their walking quantity and quality were determined. Pressure sensors are effective for recording the changes in the plantar pressure of the human body during walking. It is an ideal assessment tool for postural stability. The study in [15] assessed fall risk of workers in the construction industry by changes of biomechanical gait stability features based on wearable insoles with pressure sensors. According to these reviewed articles, accelerometers are the most frequently used and practical wearable sensor category.

The human walking process can be regarded as several consecutive repetitive gait cycles. Generally, a gait cycle refers to the process of walking from the same foot’s toe-off/heel-strike to the next toe-off/heel-strike. A gait cycle can also be divided into a swing phase and a stance phase. This means that each cycle can be recognized by detecting the flag events like heel-strike in the gait process, the gait motion can be segmented, and the features can be further extracted. Take a gait cycle of the right foot as an example. The time from the first toe off (right Toe-Off) to the first heel landing on the ground (right Heel-Strike) is the swing phase of the right foot. Then the right foot supports the weight, and the left foot enters the swing phase. It is the stance phase of the right foot until the next right Toe-Off. To detect the flag gait events, peak detection algorithms are useful [16,17]. They achieve this function by detecting repeated peaks of acceleration or angular velocity during gait.

Kinematic data of the human body is useful for knowing about gait status and fall risk assessment. It is usually obtained by inertial sensors. However, there are some types of errors in practice, which makes it challenging to obtain accurate motion information. The mounting error is an important error in applications of inertial sensors due to the misalignment from the inertial frame (sensor coordinate frame) and the global frame (body frame). Chen et al. [16] proposed a method for mounting error calibration. The method can determine the orientation of inertial frame with respect to the global frame. The results showed that it corrected the mounting error greatly. The integration drift is another kind of common errors in practice of inertial sensors. It comes from the accumulated signal noise in the process of integrating acceleration into velocity or angular velocity into angular displacement. Filters like Butterworth filter [18], Kalman filter [19] are usually used to eliminate the integration drift.

### 2.2. Non-Wearable Sensors

Pressure sensors can be either wearable or non-wearable. In addition to embedding to insoles or shoes, pressure sensors also can be used in treadmill or pressure platform like Wii balance board [20].

Infrared sensors and laser sensors are the most frequently used ones of non-wearable sensors. Nishiguchi et al. [21] developed a device that was based on an infrared laser sensor (laser range finder) to assess stepping performance. Further a new index “stepping response score” was created to assess fall risk of community-dwelling elderly individuals. The infrared laser sensor in this research was used to measure spatial and temporal parameters of steps by detecting position and motion of both legs.

Microsoft Kinect is often used in gait analysis and further for fall prevention. It consists of RGB cameras and infrared sensitive cameras and can produce depth images. Dubois et al. [22] proposed a system based on Microsoft Kinect camera to help preventing falls of the elderly people. They extracted three gait spatiotemporal features from the vertical displacement of the center of mass of the subject. The features are step length, step duration and gait speed. The features were compared to those obtained by the carpet. The results showed that their approach of gait analysis was effective. However, they did not further use this approach for fall risk assessment. Stone et al. [23] compared gait measurements by Kinect to those using a web-camera based system and those from a Vicon motion capture system. The results showed good agreements among them and confirmed the effectiveness of Kinect for passive fall risk assessment.

Infrared or laser sensors have the advantage of being precise. However, they suffer from many other issues. For example, the clothes worn by the subjects may affect the reflection of infrared rays, and multiple devices may be required due to limited field of view of sensors. This will raise the cost. Moreover, it usually requires participants to walk within a limited area which it can see. Compared to this kind of sensor, wearable sensors are cheaper and easier to deploy. Furthermore, wearable sensors are more flexible whether they are directly secured on body or embedded in clothes. Nevertheless, wearable sensor-based fall risk assessment still suffers from issues like size, battery, and data transmission, to name but a few.

In the procedure of fall risk assessment, participants will be required to do assessment tasks. The most commonly used one is steady walking on the treadmill or ground. In addition, the Timed Up and Go (TUG) test [24] is often combined with sensors to assess fall risk. It measures the time it takes for the elderly to stand up and walk and then come back and sit down. The traditional TUG test is single-task. Research has found that the dual-task test results are better [25]. The five-times-sit-to-stand test (FTSS) is another test used for assessing fall risk. The time to finish this test is measured to indicate fall risk [13].

## 3. Sensor-Based Features for Fall Risk Characterization

In sensor-based fall risk assessment, features are extracted from sensor signals to quantify one’s gait or posture characteristics. For most studies, it is necessary to identify each gait cycle by algorithms. Then the measurement values are calculated for each step. For instance, the gait speed of each step can be obtained by integrating the acceleration value obtained from accelerometers. Our survey do not focus on algorithms for identifying gait cycles or raw signals from sensors but concentrates on features extracted finally. In the following we summarize the common features according to their described gait characteristics.

### 3.1. Gait Intensity

Number of steps in a period is often used to reflect whether the participant is vigorous over a period of time. Cadence is the value of the number of steps divided by walking time. It reflects the intensity of gait. Too low or too high cadence during walking indicates abnormal gait patterns. Too low cadence may be related to freezing gait, and too high cadence may be related to festinating gait. These two abnormal gait patterns have been identified as highly correlated to fall incidents [26,27].

### 3.2. Gait Variability

Time-related features are useful in fall risk assessment. They include step time, stride time, stance time, and swing time during walking, single support time, double support time and so forth. Gait variability refers to the fluctuation in the value of a feature from one step to another [28]. These time-related features can quantify gait variability by computing average variance or standard deviation or coefficient of variation of them [29]. Hausdorff et al. [30] used the standard deviation of each participant’s stride time and swing time to quantify gait variability. They placed force-sensitive insoles in participants’ shoes to identify each stride. Then those required time-related features are determined for each stride by algorithms. The gait variability was further quantified by calculating the standard deviation of those features. The results showed that these two measures of gait variability were predictive of future falls, and the possibility of falling is positively correlated with the degree of gait variability. Generally, higher variability of gait means higher fall risk [31]. In addition to time-related features, trunk acceleration is also suitable for quantifying the variability of human gait [32]. The authors in [32] used standard deviation of trunk acceleration to measure gait variability under inclined conditions.

### 3.3. Gait Stability

Stability of gait is another important indicator for assessing fall risk. Gait stability is close to gait variability but not equal to it. Gait stability refers to the ability of maintaining gait stable when walking under small perturbations or recovering from an external perturbation. Hollman et al. [33] used variability of velocity from stride to stride to quantify gait stability. GAITRite is responsible for measuring the spatiotemporal parameters required for this study. The study was to examine whether gait stability differ in older adults compared with young adults during normal walking and walking while performing cognitive tasks. The results indicated that variability of velocity from stride to stride was greater during dual task walking, and dual task walking had a larger impact on the older adults than young adults. Therefore, walking with cognitive tasks may increase the gait instability and risk of falls. In addition to linear statistics, non-linear measures like maximum Lyapunov exponent (λs) are also used in stability assessment [32]. Local dynamic stability (LDS) is based on λs, which can be calculated by Rosenstein’s algorithm [34]. LDS is a nonlinear parameter which is derived from dynamic system theory to assess gait stability. There is a negative correlation between LDS and maximum Lyapunov exponent. When λs increases, LDS decreases and fall risk increases.

### 3.4. Postural Stability

Postural stability refers to the ability of maintaining body stable when standing. Melzer et al. [35] measured the postural stability of subjects in wide stance and narrow stance to find differences between fallers and non-fallers. The features were based on center of pressure (COP), which included COP path length, elliptical area, COP velocity, medio-lateral (ML) sway length, and antero-posterior sway length. The results indicated that there were no significant differences between two groups when standing in wide stance. Significant differences emerged when subjects standing in narrow stance. Fallers had significant higher values of COP path length, elliptical area, COP velocity, and ML sway length in various conditions which included eyes open, eyes closed, and eyes open while standing on the foam.

### 3.5. Gait Symmetry

Gait symmetry reflects the control of the lower limbs on both sides during walking. Jiang et al. [36] pointed out that gait symmetry as well as gait stability is important for fall risk assessment. There are four frequently used methods to measure gait symmetry, namely symmetry ratio, symmetry index, gait asymmetry, and symmetry angle [37]. They are showed in Table 1. Symmetry ratio index has been used in clinical measurement of gait symmetry but has a low sensitivity [38]. The symmetry index is a symmetry evaluation standard based on ground reaction forces proposed by Robinson et al. [39]. Gait asymmetry is a logarithmic transform of symmetry ratio. In [40], the authors evaluated the degree of asymmetry by comparing the swing time of the legs on both sides. Symmetry angle was proposed by Zifchock et al. [41]. Zifchock certified that symmetry angle is highly correlated with symmetry index. This suggest that symmetry angle may be a good substitute for symmetry index.

Among these four measures, there is no recognized standard for assessing gait symmetry. Patterson et al. [37] analyzed and compared these four measures for stroke patients and normal people. Stroke patients were at high risk of falls. They used five features including step length, swing time, stance time, double support time and ratio of swing time to stance time in the above four equations respectively. Analysis results suggested that no equation performed better in distinguishing stroke patients. On the contrary, different gait features have a more significant impact on the results. However, symmetry ratio may be more interpretable than the others. Therefore, the authors recommended symmetry ratio as a candidate standardization.

### 3.6. Gait Smoothness

Gait smoothness is associated with the quality of walking control. It reflects the continuousness of walking. It is usually measured by harmonic ratio. Harmonic ratio is a frequency feature in fall risk assessment. It is the ratio between the sum of the magnitudes of the even to the odd harmonics over a single stride. A higher value of harmonic ratio indicates smoother gait when walking. Parkinson’s patients and stroke patients usually perform poorly in smoothness. Low smoothness may lead to falls. Doi et al. [42] used harmonic ratio of trunk acceleration to predict falls of elderly based on prospective research method. In this study, researchers calculated the harmonic ratio of acceleration of upper and lower trunk by digital Fourier transformation in each direction. The results indicated that the harmonic ratio of upper trunk acceleration was independently associated with incidence of falls in a year. It was confirmed by ROC curve analysis that the harmonic ratio of upper trunk acceleration had high specificity for predicting potential future falls.

## 4. Fall Risk Modeling and Assessment Approaches

### 4.1. Conventional Machine Learning

The mainstream modeling approaches in sensor-based fall risk assessment are related to machine learning techniques. Conventional machine learning approaches for fall risk modeling and assessment can be classified into two categories: discriminative and generative. Discriminative models are to find a decision boundary through which samples are divided into corresponding categories. Discriminative models mainly include linear regression, logistic regression, linear discriminant analysis, Support Vector Machine (SVM), to name but a few. Generative models are to learn the boundary of each category instead of the single decision boundary. Generative modeling approaches include Naïve Bayesian classifier, k-Nearest Neighbor (KNN), Dynamic Bayesian Network (DBN) and so on. In the following, we cover the frequently used conventional machine learning approaches in sensor-based fall risk assessment.

#### 4.1.1. Discriminative Modeling Approaches

Perhaps the most frequently used discriminative modeling approach is regression which includes linear regression and logistic regression. Linear regression is a regression analysis method that uses linear regression equations to model the relationship between independent variables and dependent variables. Liu et al. [43] used multiple linear regression to map features which derived from accelerometer data to the number of falls in the past one year. In this research, a triaxial accelerometer was mounted on participants’ waist. There were 126 features extracted from the acceleration data of 68 subjects and a discriminant classifier was established according to an applied threshold value. The classifier obtained an accuracy of 97% in identifying multiple-fall fallers, and the accuracy in estimating the number of falls in the last year was 71%. This study is a retrospective analysis to explore the relationship between gait status and fall history. Therefore, its result cannot be directly used in predicting future falls. Nevertheless, it may be used as a reference in prospective studies.

Compared to linear regression, logistic regression is more commonly used. Logistic regression converges the output range from the real number domain to [0, 1] through sigmoid function. It is used in binary classification problem and more robust than linear regression. Doheny et al. [13] applied the five-times-sit-to-stand test to obtain acceleration data by an accelerometer attached to the lateral thigh to identify each sit-to-stand-to-sit phase and sit-to-stand and stand-to-sit processes. Another accelerometer was attached to the sternum to capture trunk acceleration. Participants were 39 elderly people, 19 of whom had a history of falls. Logistic regression was used to classify the participants based on their status. There were totally 70 accelerometer-derived features which were the mean and variation of the root-mean-squared amplitude, jerk and Spectral Edge Frequency (SEF) of the acceleration during each section of the assessment. Four features were finally selected for modeling based on test-retest reliability of each feature. Their model’s accuracy was 74.4%, specificity was 80.0% and sensitivity was 68.7%. It is worth mentioning that the number of participants was relatively small. It may lead to overfitting of the model. 

Support Vector Machine is another discriminative modeling technique which is a kind of generalized linear classifier for binary classification. SVM finds a hyperplane to classify two categories, and the hyperplane needs to be as far away as possible from the nearest element of each category. Greene et al. [44] applied a body-worn inertial sensor (SHIMMER) which was attached to the lower back of participants and pressure data was obtained by a Tactex S4 HD pressure mat. The root mean square amplitude of the medio-lateral and the anterior-posterior acceleration was measured for quantifying postural sway of each direction. For frequency domain, the spectral edge frequency and the spectral entropy (H) were calculated for acceleration and angular velocity signals. SVM was used to distinguish between fallers and non-fallers and obtained a mean classification accuracy greater than 70%. The result was better than that of using Berg Balance Scale (BBS) as a comparison.

Discriminative approaches are used to model conditional probability and find the optimal boundary between different categories. It pays more attention to the differences between different categories than the characteristics of the sample data itself. Compared to generative modeling, it can work by less computing resource and samples and has better predicting performance in most practical cases.

#### 4.1.2. Generative Modeling Approaches

Naïve Bayesian classifier may be the simplest generative modeling approach. It has been used with satisfying results for fall risk assessment [11,12,45,46]. Naïve Bayesian classifier is based on Bayes theorem and assumes that the features are conditional independent of each other. This assumption will lead to the decline of classification accuracy when the correlation among features is large. K-Nearest Neighbor is another generative modeling approach that can be used in fall risk assessment [46,47]. KNN method determines the category of the samples to be divided according to the category of the nearest one or several samples. The disadvantage of KNN is the large amount of calculation, because for each sample to be classified, the distance from itself to all known samples must be calculated.

Bayesian Network (BN) is a type of graphical model to describe the dependency relationship between data variables. Dynamic Bayesian Network is an extention of BN. It can represent the evolution of variables over time. Cuaya et al. [48] built two DBN models for predicting falls in next six months. One feature set was established under the guidance of experts from the Human Motion Analysis Laboratory of the INR. Another one was founded on the feature set automatically selected by forward sequential selection (FSS) algorithm. The expert-guided model showed slight advantages over the model applying FSS. However, considering the small sample size, a conclusion that expert-guided feature selection is better than FSS cannot be made.

### 4.2. Deep Learning

Neural networks are based on perceptron, so neural networks are sometimes called multi-layer perceptron, namely Artificial Neural Networks (ANN). Deep learning is the general name of pattern analysis methods based on ANN. The neural network layer in ANN can be divided into three categories: input layer, hidden layer and output layer. Generally speaking, all middle layers are hidden layers. Basically, deep learning approaches can be divided into two categories: supervised learning and semi-supervised/unsupervised learning. However, most studies of fall risk assessment using deep learning are based on supervised learning. There are few studies using semi-supervised or unsupervised learning approaches. Therefore, we focus on the applications of supervised learning approaches in fall risk assessment.

Deep neural networks (DNN) are composed of a large number of simple processing modules named “neurons”. These “neurons” are distributed in separate layers and their common task is calculating the “activation function” of the weighted sum of their inputs. DNNs have the ability to learn directly on the raw data, so as to reduce the demand for feature engineering. Full connected DNNs are suitable for most classification tasks theoretically. However, they are rarely used in practice due to demand for large amount of data.

Nait Aicha et al. [49] used a dataset which consists of acceleration data from 296 older people. They compared Convolutional Neural Networks (CNN), Long Short-Term Memory (LSTM), a combination of them and a base model which used biomechanical features in single-task learning and multi-task learning respectively. The results indicated that deep learning methods perform better in identifying subjects and assessing fall risk with gender and age as auxiliary tasks when doing multi-task learning.

Recurrent Neural Networks (RNN) are neural networks with sequence data as input. LSTM and Bi-directional long short-term memory (BiLSTM) are the most common RNNs for studies of fall risk assessment. Meyer et al. [50] analyzed a variety of machine learning models and feature sets for fall risk assessment of patients with multiple sclerosis. For conventional machine learning methods which were logistic regression, SVM, decision tree, KNN and ensemble binary statistical classification models in this study, feature sets manually calculated from accelerometer data were used. Deep learning methods do not require features to be manually calculated. Deep leaning models can take raw data as input, extract features automatically and finish the classification task. In this study, BiLSTM which combines forward LSTM with backward LSTM was used. BiLSTM is based on Recurrent Neural Network. In discriminating patients with a fall history from those without, BiLSTM obtained an accuracy of 86% and an AUC of 0.88. It performed better than all conventional machine learning methods used in this study. It is worth mentioning that BiLSTM may be better than LSTM when considering retrospective fall status classification as an intermediate step in prospective fall risk assessment.

Recurrent Neural Networks are suitable for sequence data, which leads to frequent use. Tunca et al. [51] explored LSTM for fall risk assessment as well. LSTM is sequence-to-label classifier that can operate on sequence data directly. Considering that the existing research on fall risk assessment and gait analysis has accumulated valuable domain knowledge, this study attempted to combine the domain knowledge inherent in the spatio-temporal gait features with LSTM. Sequences of spatio-temporal gait features from a sensor-based system were used as input. Data samples consisted of four-dimensional sequences whose length are ten. Four dimensions were stride length, clearance, stance time and swing time. The length was due to 10 strides in a window which was used for data augmentation. In addition to sequence data, another LSTM model was trained by raw data to determine whether the model can implicitly learn the required features. The results showed that LSTM with sequences of gait features achieved an accuracy of 89% on a validation set and 92.1% on a separate test set.

Convolutional Neural Networks are feedforward neural networks with convolutional calculation. Although most applications of CNNs are related to pictures, they can handle most grid-like data. Time series data commonly used in fall risk assessment can be considered as one-dimensional grid-like data. Savadkoohi et al. [52] used a one-dimensional CNN with force plate time series data. Their network consists of three convolutional layers, a max pooling layer and a global average pooling layer. The authors used only two convolutional layers before the max pooling layer to minimize the information loss. Global average pooling was used to reduce the possibility of overfitting.

### 4.3. Knowledge-Driven Model

In addition to data-driven models mentioned in above two sections, there have been a small quantity attempts to perform fall risk assessment by knowledge-driven models. Farseeing Fall Risk Assessment Tool (FRAT-up) introduced in [53] is based on probabilistic rules, generated automatically from a light ontology. FRAT-up is based on an assumption that the total fall risk of a person is determined by the contributions of the risk factors related to falls. The system takes the characteristics of a subject in terms of risk factors. As output FRAT-up provides an estimation of the fall risk.

Compared to data-driven models, knowledge-driven models may be not applicable to sensor-based fall risk assessment.

A comparison between different fall risk modeling approaches is showed in Table 2.

## 5. Challenges

This survey analyzed literatures about sensor-based fall risk assessment, including sensors themselves, features extracted from sensor data and modeling approaches. There are still some challenges needed to focus on and they are listed below.

### 5.1. Optimal Sensor Placement

Different parts of human body show different motion characteristics in the process of walking. Fixing sensors on various body parts can obtain various types of data and extract various gait features. For example, sensors attached to thighs can monitor the process of sit-to-stand. Many body positions have been tested. However, researchers have not reached a consensus on the optimal position of sensors for the work of assessing fall risk.

### 5.2. Better Task for Risk Assessment of Falling

In the step of motion monitoring and data collecting of fall risk assessment, subjects are required to perform a task. It may be walking on flat ground, walking on the slope, Five-Times-Sit-to-Stand. In addition to single-task, dual-task walking has been used in some studies. However, dual-task walking does not significantly help to improve the performance of the model for fall risk assessment. It is important to find tasks that can better characterize human gait.

### 5.3. Insufficient Sample Size

In most studies we reviewed, the sample size is usually small. Many studies included no more than 100 subjects, and due to the requirement of long and intensive follow-up period, the final sample size may be smaller. In addition, the continuous tracking of a large number of subjects is also a costly work. Too little total sample size may lead to overfitting of the final model. Too few positive samples relative to total sample size may lead to distorted models.

## 6. Future Directions

### 6.1. More Robust Feature Construction

Feature selection is a commonly used approach of feature dimensionality reduction. It can simplify our final model and reduce the overfitting of model. Compared with automated feature selection, the extraction of raw feature set depends more on manually operating. Therefore, more effective feature selection approaches are important for feature construction before modeling. In addition, knowledge in other fields like physiatry may help find the categories of raw features which are more closely associated with falls. Most studies today focus on motion signals of human. In fact, physiological signals can also reflect human’s status. Feature construction based on physiological signals may be also a future direction.

### 6.2. Public Database of Various Datasets

Based on the extensive literature search, we found that the studies focus much on the way sensors are placed and how to design tasks before building a model. Authors acquire data in various ways: Types and position of sensors, tasks that paticipants should do, sampling frequency, extracted features, to name but a few. Building a public database with these various datasets acquired by different ways could help compare and reuse the results.

### 6.3. Daily-Life Monitoring System

Most studies are carried out in laboratories or clinical environments. Professionals are in need and data acquisition is inflexible. Moreover, participants who go to lab may feel nervous when doing the task required so that they show a motion pattern which is different from the usual one. Therefore, developing an daily-life continuously gait monitoring system is important. In order to make elderly people continuously use the gait system, it cannot be intrusive and clumsy, and user-friendly design should be under consideration to make users comfortable with it. The wireless Inertial Measurement Unit (IMU) is a good choice due to its small size and convenience of data transmission. Embedding sensors into daily clothes is a new developing direction. Rosa et al. [54] developed an electric insole based system. It transferred data collected to user’s smartphone in real time. And the smartphone further transferred the data to backend server to analyze. The smartphone itself also can be used for fall risk assessment due to the sensors it contains. Nishiguchi et al. [55] developed a mobile phone application to validate its capacity to quantify gait features. However, there are issues when using smartphone as the tool to assess fall risk. For example, people carry their smartphones in different places (bags, pockets, etc.), which may affect the assessment process of mobile phone.

## 7. Conclusions

Fall risk assessment has become a promising direction in the health industry. The development of various disciplines, e.g., machine learning, sensor networks and wireless communications, has jointly promoted the progress in this field. In this work, a survey of fall risk assessment based on sensors has been presented. We first introduced principles and methodology of this field. Then we reviewed monitoring, modeling and assessment of fall risk respectively. In particular we identified characteristics of above aspects and reviewed each individual field by category. At last, we discussed the existing challenges and promising directions in this field.

## Figures and Tables

**Table 1:** Calculation formula of gait symmetry.

Measurement	Abbreviation	Calculation Formula
Symmetry ratio index	RI	$\left(1-\frac{x_{r}}{x_{l}}\right)*100\%$
Symmetry index	SI	$\frac{\left|x_{r}-x_{l}\right|}{0.5\left(x_{r}+x_{l}\right)}*100\%$
Gait asymmetry	GA	$\ln\left(\frac{x_{r}}{x_{l}}\right)*100\%$
Symmetry angle	SA	$\frac{45{}^{\circ}-\tan^{-1}(\frac{x_{r}}{x_{l}})}{90{}^{\circ}}*100\%$

$x_{r}$ and $x_{l}$: values of specific features for right and left limbs.

**Table 2:** The comparison of fall risk modeling approaches.

	Conventional Machine Learning	Deep Learning
Model type	Logistic regression, SVM, KNN, HMM	CNN, LSTM, BiLSTM
Advantage	More friendly to small sample size, cheaper, more interpretable	Higher accuracy, no need for engineering
Disadvantage	Lack of scalability	More expensive, hard to understand

## Data Availability

Not Applicable.
